# Personality traits, coping styles, and perceived stress across polish military formations and a civilian comparison group

**DOI:** 10.3389/fpubh.2026.1890043

**Published:** 2026-10-08

**Authors:** Marta Kopańska, Danuta Ochojska, Patrycja Leśnicka, Izabela Sarzyńska, Krzysztof Przednowek, Rafał Podgórski

**Affiliations:** 1 Department of Medical Communication and Professional Competency Development, Faculty of Medicine, University of Rzeszów, Rzeszów, Poland; 2 Faculty of Health Sciences and Psychology, Medical College, University of Rzeszów, Rzeszów, Poland; 3 Student Research Club “Reh-Tech”, Collegium Medicum, University of Rzeszów, Rzeszów, Poland; 4 Faculty of Physical Culture Sciences, Medical College of Rzeszów University, Rzeszów, Poland; 5 Department of Medicinal Chemistry and Metabolomics, Faculty of Medicine, Medical College of Rzeszów University, Rzeszów, Poland

**Keywords:** coping strategies, mental health, neuroticism, personality traits, psychological stress, soldiers, special forces

## Abstract

This cross-sectional study examined differences in personality traits, coping styles, and perceived stress across three Polish military formations and a civilian comparison group, and associations of personality traits and coping styles with perceived stress. The sample comprised 193 men: 37 Special Forces soldiers, 54 Regular Army soldiers, 48 Territorial Defence Forces (WOT) soldiers, and 54 civilIians without military service experience. Participants completed the NEO-FFI Personality Inventory, the Coping Inventory for Stressful Situations (CISS), and the Perceived Stress Scale (PSS-10). The groups differed in selected personality traits, coping styles, and perceived stress. Mean PSS-10 scores were 11.16 in Special Forces, 16.04 in civilians, 15.31 in the Regular Army, and 14.98 in WOT. Special Forces soldiers showed higher conscientiousness than all other groups, lower neuroticism than civilians, and more frequent task-oriented coping than civilians. Neuroticism (*r* = 0.59) and emotion-oriented coping (*r* = 0.60) showed the strongest positive bivariate associations with perceived stress within their respective domains. The findings describe differences between the sampled groups rather than a uniform military–civilian distinction. They do not establish effects of selection, training, or operational experience. Incomplete demographic and occupational characterization limits between-group interpretation. Longitudinal studies with better-characterized comparison groups are needed to examine the implications of these associations for stress-management support.

## Introduction

Military service encompasses occupational contexts that differ in their duties, organization, and demands. Work-related stress, operational readiness, responsibility, and deployment-related separation from family have been discussed as sources of psychological burden in military populations (1–3). However, belonging to a military formation does not establish an individual’s exposure to a particular stressor or the level of stress that the individual perceives. Distinguishing service context from perceived stress is therefore important when comparing military and civilian participants.

Previous research has often focused on specialized operational units or combat-deployed personnel (4–6). However, military service includes formations with different organizational arrangements and service roles (7–9). The present study examines personality traits, coping styles, and perceived stress in Special Forces, the Regular Army, and the Territorial Defence Forces, alongside civilian men without military service experience. This design allows us to examine whether the observed psychological profiles are shared across the sampled military groups, are more specific to particular service contexts, or differ from the civilian reference group.

From the interactional perspective described by Lazarus and Folkman, stress concerns the relationship between individuals and environmental demands as they appraise those demands and their available resources. Primary appraisal concerns whether a situation represents a threat, loss, or challenge, whereas secondary appraisal concerns the resources available to respond to it (10). This perspective provides a rationale for considering individual characteristics, coping styles, and perceived stress together, without assuming that formation membership alone determines the subjective experience of stress.

Personality traits, coping styles, and perceived stress describe complementary rather than interchangeable aspects of psychological functioning. In this study, the NEO-FFI assesses personality traits, the CISS assesses reported coping styles, and the PSS-10 assesses subjectively perceived stress during the preceding 4 weeks. Research on coping and emotion regulation provides a context for examining how reported ways of responding to difficulties are associated with perceived stress (11, 12). Assessing personality traits, coping styles, and perceived stress together allows complementary aspects of psychological functioning to be examined. Personality traits describe relatively stable dispositional characteristics, coping styles describe reported cognitive and behavioral responses to stressors, and perceived stress captures the subjective experience of stress. This joint assessment allows group profiles and associations with perceived stress to be characterized, but it does not establish temporal order or mediation (13, 14).

The three military groups represent different service contexts: specialized operational duties in Special Forces, regular service in a land-force formation, and territorial service combining military and civilian roles. The civilian group provides a non-military reference group assessed with the same instruments. Including all four groups allows the study to examine whether the observed profiles are shared across military formations, are more specific to particular service contexts, or differ from the civilian reference group. None of the groups was treated as representing a predefined level of stress exposure, and civilian participants were not assumed to represent a low-stress population.

The present cross-sectional study examined personality traits, reported coping styles, and perceived stress among men serving in the Special Forces, the Regular Army, and the Territorial Defence Forces, and civilian men without military service experience. The central research question was whether these groups differed in personality traits, reported coping styles, and perceived stress. A complementary objective was to examine associations of personality traits and coping styles with perceived stress across the study sample. Because individual exposure to stressors and occupational histories were not comprehensively characterized, the study does not test the effects of combat exposure, selection, or military training.

## Materials and methods

The study had a cross-sectional design and included 193 men in Poland from three military formations and a civilian comparison group. Only male participants were included because women represented a very small proportion of the personnel available in the participating military formations. Restricting the sample to men was intended to maintain a sufficiently homogeneous sample for the planned comparisons, but limits generalizability to women. Participants were recruited purposively into the following groups (Table 1):

- Special Forces (SF): personnel of combat subunits specializing in high-risk operations. This description characterizes the formation and does not constitute an individual measure of selection history or operational exposure.
- Regular Army (RA; 21st Podhale Rifles Brigade): soldiers of a light infantry formation carrying out tasks in specific terrain and climatic conditions.
- Territorial Defence Forces (WOT): soldiers in territorial military service, combining military service with professional activity in the civilian environment.
- Civilian comparison group (CG): men without a history of military service, recruited as a non-military reference group rather than as an established demographically or occupationally matched control group.

Active military service was required for inclusion in the military groups. All military participants performed active service duties within their respective formations and were not employed exclusively in administrative roles. Individual data on length of service, combat experience, and previous traumatic experiences were not collected. Descriptions of the formations therefore provide organizational context, not evidence that participants had comparable professional trajectories, training, or exposure to stressful events. The contribution of these unmeasured characteristics to the observed group differences cannot be determined.

**Table 1:** Number of participants in each study group.

Group	*N*	Age, years
Special forces (SF)	37	35.0 (32.0–39.0)
Civilian comparison group (CG)	54	34.0 (28.0–40.0)
Regular army (RA)	54	30.5 (26.0–36.0)
Territorial defence forces (WOT)	48	33.0 (26.0–39.0)

Age is presented as median (Q1–Q3). Q1 and Q3 denote the first and third quartiles, respectively. SF, Special Forces; CG, civilian comparison group; RA, regular army; WOT, Territorial Defence Forces.

Median age ranged from 30.5 to 35.0 years across the four groups. Participants were therefore generally in early to middle adulthood, and the group medians were descriptively similar.

The civilian participants were included as a non-military comparison group without military service experience. Their inclusion allowed the study to examine whether the psychological profiles observed in the military groups were shared across formations or differed from those of the civilian participants recruited into the study. Absence of military experience was not treated as evidence of low stress exposure. Because civilian occupational characteristics, work demands, and other sources of stress were not sufficiently characterized, this group cannot be regarded as an occupationally or exposure-matched, low-stress reference population. The common eligibility criteria also do not establish demographic equivalence between groups.

### Inclusion and exclusion criteria

The inclusion and exclusion criteria applied to all groups are summarized in Table 2. These criteria defined eligibility but do not, by themselves, eliminate confounding or establish comparability of the groups.

**Table 2:** Inclusion and exclusion criteria.

Inclusion criteria	Exclusion criteria
Active-duty soldier (professional or territorial service) or no history of military service (civilian group)	Diagnosed neuropsychiatric or neurological disorders
Male, aged 18–50 years	Active substance or alcohol dependence
Written informed consent	Incomplete questionnaires (NEO-FFI, CISS, PSS-10)

Eligible participants were men aged 18–50 years belonging to one of the military groups or, for the civilian group, without a history of military service. This age interval was an eligibility criterion, not a measure of the observed age distribution within each group. Participation required voluntary written informed consent and completion of the full set of psychological questionnaires.

Individuals with diagnosed neuropsychiatric or neurological disorders that could affect the results of self-report measures were excluded from the study. An additional exclusion criterion was active addiction to alcohol or psychoactive substances, as declared in the medical or occupational interview. Participants who failed to correctly complete at least one of the key research instruments (NEO-FFI, CISS, or PSS-10) were also excluded from the analysis.

### Research instruments

The study employed standardized psychological instruments designed to assess three main areas of psychological functioning: personality traits, the level of perceived stress, and stress coping styles.

### NEO-FFI personality inventory

Participants’ personality traits were assessed using the NEO-FFI Inventory, which is a shortened version of the NEO PI-R Personality Inventory. It is used to assess five basic dimensions of personality: neuroticism, extraversion, openness to experience, agreeableness, and conscientiousness. The instrument consists of 60 statements, with 12 items for each of the analyzed traits. Responses are provided on a 5-point Likert scale ranging from 0 (“strongly disagree”) to 4 (“strongly agree”), with higher scores indicating a greater intensity of a given personality trait. Completion of the NEO-FFI takes approximately 15 min (15, 16).

### Coping inventory for stressful situations (CISS)

Stress coping styles were assessed using the Endler and Parker CISS Questionnaire. This questionnaire is a self-report instrument designed to evaluate ways of coping in stressful situations. It consists of 48 items and enables the analysis of three basic stress coping styles: task-oriented, emotion-oriented, and avoidance-oriented coping, with each dimension comprising 16 statements. The task-oriented style refers to actions aimed at solving the problem, planning, and actively attempting to change a difficult situation. The emotion-oriented style describes a focus on one’s own emotions and psychological reactions. In contrast, the avoidance-oriented style is associated with attempts to reduce tension by diverting attention from the stressor, engaging in other activities, or seeking social contact. Responses are provided on a 5-point Likert scale ranging from 1 (“not at all”) to 5 (“very much”), indicating the frequency of engaging in particular behaviors in difficult or stressful situations. The avoidance-oriented style (SSU) also includes engagement in substitute activities (ACZ) and seeking social contacts (PKT). These component scores overlap with the broader avoidance-oriented score and are not independent coping dimensions. SSZ and SSE denote task-oriented and emotion-oriented coping, respectively.

### Perceived stress scale (PSS-10)

The level of perceived stress was measured using the Perceived Stress Scale (PSS-10). This scale measures subjectively perceived stress over the past 4 weeks. The questionnaire consists of 10 questions referring to the frequency of specific thoughts and feelings related to stress. Responses are provided on a 5-point scale ranging from 0 (“never”) to 4 (“very often”). Higher total scores indicate greater perceived stress (17). The Polish adaptations of the NEO-FFI, CISS, and PSS-10 were applied without modification. The analyses used raw scores to examine relative differences and associations across the four study groups, rather than to classify participants into low, average, or high normative categories. The group means should therefore not be interpreted as indicating low, average, or high levels relative to the Polish general population.

### Study procedure

The study had a cross-sectional design and was conducted using standardized questionnaire-based methods. All protocols implemented in this study were approved by the Ethics Committee of the University of Rzeszów, Poland (resolution 2023/12/0060 of 6/12/2023), and were performed following the Declaration of Helsinki. Informed consent from participants was signed before data collection. Participants were recruited within individual study groups, and participation in the study was voluntary.

The research procedure consisted of a one-time completion of a set of psychological instruments assessing personality traits, the level of perceived stress, and preferred coping styles in difficult situations. This procedure was conducted on-site in dedicated facilities at the University of Rzeszów. Soldiers from individual operational units (Special Forces, Regular Army, and WOT) were delegated and arrived at the academic center in order to participate in the study. The use of identical environmental conditions enabled full standardization of the data collection process and ensured optimal comfort and complete confidentiality for participants while completing the set of psychometric instruments. The course of the study was supervised each time by the research team, which guaranteed the correctness and reliability of the collected materials. Before participation, all individuals were informed about the aim of the study, its anonymous nature, and the possibility of withdrawing at any stage without providing a reason. Written informed consent was obtained from each participant prior to participation in the study.

### Statistical analysis

Descriptive statistics (means, standard deviations, and medians) were calculated for the psychological variables, and the normality of distributions was assessed using the Shapiro–Wilk test. Group differences were examined using one-way analysis of variance (ANOVA) with Welch’s correction for variables with distributions close to normal, and the Kruskal–Wallis test for variables that did not meet parametric assumptions. Post-hoc pairwise comparisons were conducted, including Dunn’s test with Bonferroni correction for non-parametric analyses. Relationships between perceived stress, personality traits, and coping styles were analyzed using Pearson’s correlation coefficient (r). Statistical significance was set at *p* < 0.05, and partial eta-squared (η^2^p) was reported for the relevant models. Raw-score comparisons describe the sampled groups and do not establish normative categories or statistical equivalence.

Linear regression models were used to examine associations with PSS-10 scores conditional on the included psychological variables and group membership, with Special Forces as the reference group. Separate models included personality traits and coping scores. Age was not included as a covariate; group coefficients therefore cannot be interpreted as age-adjusted associations. The term predictor denotes a variable in a statistical model and does not imply temporal prediction or causation.

The original coping specification included group membership, the three main CISS styles (SSZ, SSE, and SSU), and both avoidance components (ACZ and PKT). This overlapping specification is reported as exploratory. Its component coefficients describe associations conditional on the avoidance total and the other included scores, rather than separable associations of distinct coping constructs. Such overlap can increase coefficient uncertainty and sensitivity to model specification; individual coefficients are therefore not used to establish an absence of an association for avoidance coping or either component.

As a complementary analysis, the personality model and the original exploratory coping model were estimated within a Bayesian framework using Bayesian model averaging implemented in the BAS algorithm (jsq module, jamovi), with a Jeffreys–Zellner–Siow prior on regression coefficients and a uniform prior over the model space. The reported inclusion probabilities and Bayes factors are conditional on these candidate predictor sets and priors. Bayesian model averaging does not, by itself, remove the interpretive problem caused by including an avoidance total alongside its components.

## Results

### Intergroup differences in personality traits

One-way ANOVA with Welch’s correction identified group differences in openness (*F*(3, 98.92) = 4.89; *p* = 0.003), neuroticism (*F*(3, 96.77) = 4.75; *p* = 0.004), and conscientiousness (*F*(3, 98.41) = 11.28; *p* < 0.001; Table 3). The omnibus tests for extraversion (*F*(3, 98.27) = 2.61; *p* = 0.056) and agreeableness (*p* = 0.166) did not meet the prespecified significance threshold. Special Forces had the lowest mean neuroticism and highest mean conscientiousness within the sample; the specific supported pairwise differences are described below.

**Table 3:** Differences in personality traits between groups.

Variable	SF (M ± SD)	CG (M ± SD)	RA (M ± SD)	WOT (M ± SD)	F	*p*	η^2^p
Openness	29.86 ± 7.51	30.46 ± 7.06	27.33 ± 6.19	26.15 ± 5.53	4.89	0.003	0.01
Neuroticism	11.03 ± 9.56	16.26 ± 6.29	12.70 ± 7.28	12.75 ± 5.19	4.75	0.004	0.05
Agreeableness	33.22 ± 5.91	30.94 ± 5.45	30.52 ± 5.46	31.44 ± 5.02	1.73	0.166	0.01
Extraversion	33.84 ± 6.24	30.70 ± 5.19	31.54 ± 4.78	30.56 ± 5.11	2.61	0.056	0.00
Conscientiousness	44.03 ± 6.79	36.02 ± 6.15	39.61 ± 5.15	40.10 ± 5.64	11.28	< 0.001	0.05

Results of Welch-corrected one-way ANOVA for personality traits across groups. SF, Special Forces; CG, civilian comparison group; RA, Regular Army; WOT, Territorial Defence Forces.

η^2^p - partial eta squared effect size (0.01 = small, 0.06 = medium, 0.14 = large effect).

*p* < 0.05 indicates statistical significance.

Post-hoc pairwise comparison analyses (Table 4) revealed significant differences between selected groups. In terms of conscientiousness, Special Forces obtained significantly higher scores compared to all other groups. Additionally, the civilian group was characterized by lower conscientiousness than both the Regular Army and WOT soldiers.

**Table 4:** *Post-hoc* comparisons of personality traits between groups.

Variable	Comparison	*p*-value
Conscientiousness	Special Forces vs. Civilian	<0.001
	Special Forces vs. Regular Army	0.003
	Special Forces vs. WOT	0.014
	Civilian vs. Regular Army	0.010
	Civilian vs. WOT	0.003
Neuroticism	Special Forces vs. Civilian	0.004
	Civilian vs. Regular Army	0.047
Openness	Civilian vs. WOT	0.006
	Special Forces vs. WOT	0.050
Extraversion	Special Forces vs. Civilian	0.030
	Special Forces vs. WOT	0.026

Results of *post-hoc* pairwise comparisons for personality traits.

*p* < 0.05 indicates statistical significance.

For neuroticism, Special Forces scored lower than civilians, and civilians scored higher than the Regular Army. For openness, civilians scored higher than WOT, whereas the Special Forces–WOT comparison had *p* = 0.050. Table 4 also reports pairwise extraversion comparisons. Because the omnibus extraversion test was not significant, these comparisons are treated as exploratory rather than as evidence of an established overall group difference.

### Group differences in perceived stress (PSS-10)

Welch’s ANOVA identified a difference in perceived stress across the four groups (*F* = 8.44; *p* < 0.001; Table 5). Mean PSS-10 scores were 11.16 ± 4.75 in Special Forces, 16.04 ± 4.84 in civilians, 15.31 ± 4.92 in the Regular Army, and 14.98 ± 4.48 in WOT. The descriptive pattern is a lower mean in Special Forces than in the remaining groups, whose means were relatively similar, rather than an ordered gradient across military formations. Similar group means do not demonstrate statistical equivalence, and these raw scores do not establish normative stress categories.

**Table 5:** Differences in perceived stress (PSS-10) between groups.

Variable	SF (M ± SD)	CG (M ± SD)	RA (M ± SD)	WOT (M ± SD)	F	*p*	η^2^p
PSS-10	11.16 ± 4.75	16.04 ± 4.84	15.31 ± 4.92	14.98 ± 4.48	8.44	<0.001	0.09

Results of Welch-corrected one-way ANOVA for perceived stress levels (PSS-10) across the studied groups. SF, Special Forces; CG, civilian comparison group; RA, Regular Army; WOT, Territorial Defence Forces.

η^2^p - partial eta squared effect size (0.01 = small, 0.06 = medium, 0.14 = large effect).

*p* < 0.05 indicates statistically significant differences.

### Coping styles

In order to assess differences between groups in coping styles, the Kruskal-Wallis test was applied. As presented in Table 6, statistically significant differences between groups were found for the task-oriented style (χ^2^ = 19.18; *p* < 0.001) and the emotion-oriented style (χ^2^ = 21.11; *p* < 0.001). No significant differences were observed for the avoidance-oriented style or its components, i.e., engaging in substitute activities and seeking social contact.

**Table 6:** Group differences in coping styles.

Variable	χ^2^	df	*p*
SSZ - task-oriented style	19.18	3	<0.001
SSE - emotion-oriented style	21.11	3	<0.001
SSU - avoidance-oriented style	2.58	3	0.460
ACZ - substitute activities	5.79	3	0.122
PKT - social contacts	2.37	3	0.499

Results of the Kruskal–Wallis test for coping styles.

*p* < 0.05 - statistically significant differences.

Post-hoc comparison analyses (Table 7) demonstrated that, after applying the Bonferroni correction, significant differences between groups concerned the task-oriented and emotion-oriented styles. Special Forces obtained significantly higher scores for the task-oriented style compared to the civilian group. Moreover, Special Forces were characterized by significantly lower intensity of the emotion-oriented style compared to both the civilian group and WOT soldiers. (Figure 1).

**Table 7:** *Post-hoc* pairwise comparisons of coping styles.

Variable	Comparison	*p*-value
SSZ - task-oriented coping	Special Forces vs. Civilian	<0.001
SSE - emotion-oriented coping	Special Forces vs. Civilian	0.001
	Special Forces vs. WOT	0.003

Results of Dunn *post-hoc* comparisons with Bonferroni correction for coping styles.

*p* < 0.05 indicates statistically significant differences.

**Figure 1:**
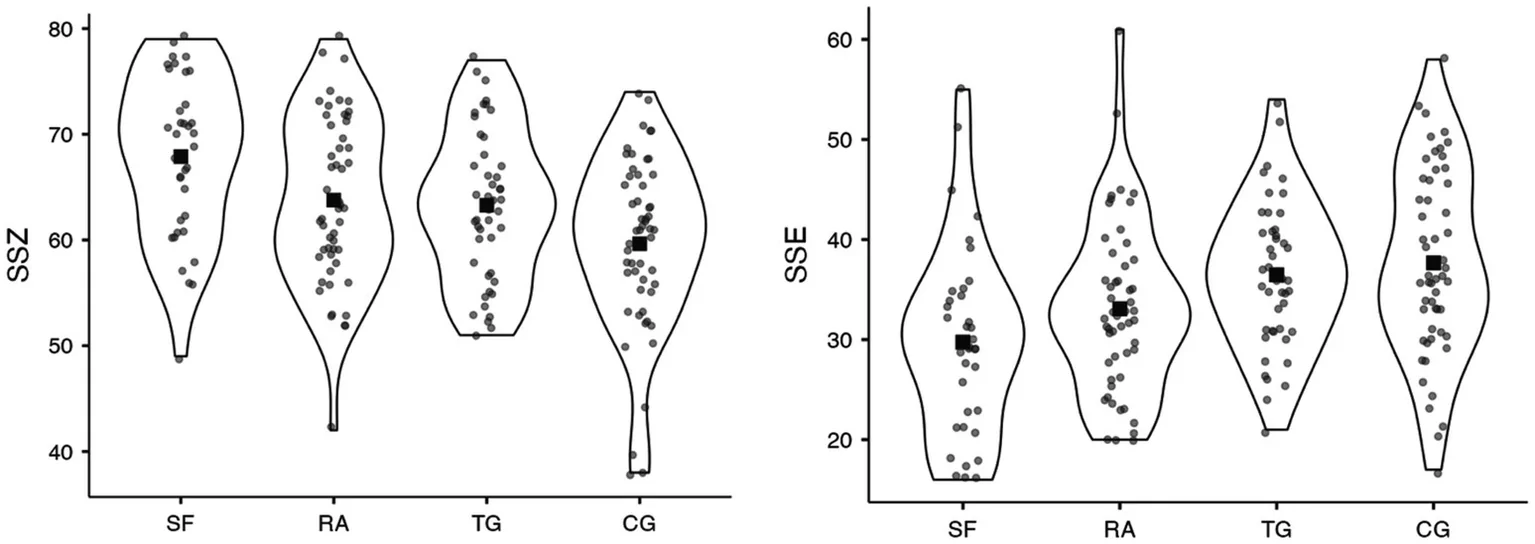
Distributions of task-oriented coping (SSZ) and emotion-oriented coping (SSE) scores across the four study groups. SF, special forces; RA, regular army; TG, territorial group (WOT); CG, civilian comparison group.

### Correlation analysis

As presented in Table 8, perceived stress (PSS-10) was significantly positively correlated with the emotion-oriented coping style (*r* = 0.60, *p* < 0.001), and to a lesser extent with the avoidance-oriented style and engagement in substitute activities. At the same time, a significant negative correlation was observed between perceived stress and the task-oriented coping style (*r* = −0.25, *p* < 0.001).

**Table 8:** Correlations between coping styles and perceived stress (PSS-10).

Variable	SSZ	SSE	SSU	ACZ	PKT	PSS-10
SSZ - task-oriented style	—					
SSE - emotion-oriented style	−0.25***	—				
SSU - avoidance-oriented style	−0.19**	0.35***	—			
ACZ - engagement in substitute activities	−0.31***	0.43***	0.89***	—		
PKT - seeking social contacts	0.14	0.07	0.71***	0.37***	—	
PSS-10 - Perceived Stress Scale (10-item version)	−0.25***	0.60***	0.18*	0.21**	0.07	—

Pearson correlation coefficients pooled across the four groups. SSZ, task-oriented coping; SSE, emotion-oriented coping; SSU, avoidance-oriented coping; ACZ, engagement in substitute activities; PKT, seeking social contacts.

*p < 0.05; **p < 0.01; ***p < 0.001.

Emotion-oriented coping was positively correlated with avoidance-oriented coping and its components. Task-oriented coping was negatively correlated with emotion-oriented coping, avoidance-oriented coping, and engagement in substitute activities, but not with seeking social contacts (Table 8). The avoidance-oriented total correlated strongly with engagement in substitute activities (*r* = 0.89, *p* < 0.001) and seeking social contacts (*r* = 0.71, *p* < 0.001), consistent with their shared score structure. The reported correlations are pooled across all four groups and do not establish identical within-group relationships.

As presented in Table 9, the level of perceived stress (PSS-10) was significantly positively correlated with neuroticism (*r* = 0.59, *p* < 0.001) and negatively correlated with extraversion (*r* = −0.28, *p* < 0.001), conscientiousness (*r* = −0.37, *p* < 0.001), and agreeableness (*r* = −0.19, *p* < 0.01). No significant relationship was found between stress and openness.

**Table 9:** Correlations between personality traits and perceived stress (PSS-10).

Variable	Openness	Neuroticism	Agreeableness	Extraversion	Conscientiousness	PSS-10
Openness	—					
Neuroticism	−0.04	—				
Agreeableness	0.13	−0.12	—			
Extraversion	0.18*	−0.36***	0.31***	—		
Conscientiousness	0.00	−0.55***	0.18*	0.40***	—	
PSS-10	−0.05	0.59***	−0.19**	−0.28***	−0.37***	—

Pearson correlation coefficients between variables.

*p < 0.05; **p < 0.01; ***p < 0.001.

Among the personality traits, the strongest relationships concerned the negative correlation between neuroticism and conscientiousness (*r* = −0.55, *p* < 0.001), as well as the positive associations of extraversion with conscientiousness (*r* = 0.40, *p* < 0.001) and agreeableness (*r* = 0.31, *p* < 0.001). Additionally, extraversion was negatively correlated with neuroticism (*r* = −0.36, *p* < 0.001).

### Regression analysis

In the personality regression model, neuroticism was positively associated with PSS-10 scores after accounting for the other personality traits and group membership (B = 0.38, β = 0.56, *p* < 0.001; Table 10). The model explained 41% of the variation in perceived stress within the analyzed sample (R^2^ = 0.41).

**Table 10:** Linear regression model predicting perceived stress (PSS-10) based on personality traits and group.

Variable	B	SE	β	*p*	η^2^p
Group					0.09
Group: Civilian vs. Special Forces	3.03	0.93	0.60	0.001	
Group: Regular Army vs. Special Forces	3.54	0.88	0.70	<0.001	
Group: WOT vs. Special Forces	3.12	0.91	0.62	<0.001	
Openness	0.00	0.05	0.00	0.973	0.00
Neuroticism	0.38	0.05	0.56	<0.001	0.27
Agreeableness	−0.02	0.06	−0.02	0.708	0.00
Extraversion	−0.06	0.06	−0.07	0.314	0.01
Conscientiousness	0.05	0.06	0.07	0.370	0.00

Linear regression model (OLS). R^2^ = 0.41.

η^2^p - partial eta squared effect size (0.01 = small, 0.06 = medium, 0.14 = large effect).

*p* < 0.05 indicates statistical significance.

Relative to Special Forces, the civilian, Regular Army, and WOT group coefficients were positive and statistically significant in this model. Openness, agreeableness, extraversion, and conscientiousness did not meet the significance threshold after conditioning on the other included predictors. These estimates are conditional associations and do not account for age or unmeasured professional experience.

In the main Bayesian coping model (Table 11), emotion-oriented coping (SSE) was the only predictor for which the data provided decisive evidence for inclusion (posterior mean = 0.30, SD = 0.03, 95% credible interval [0.24, 0.37], posterior inclusion probability = 1.00, inclusion Bayes factor > 10^12^). Higher SSE scores were associated with higher perceived stress. The data favored exclusion of the overall avoidance-oriented coping score (SSU; posterior mean = 0.00, SD = 0.02, 95% credible interval [−0.05, 0.01], posterior inclusion probability = 0.18, inclusion Bayes factor = 0.21). Evidence against inclusion was weaker for task-oriented coping (SSZ; posterior mean = −0.03, SD = 0.04, 95% credible interval [−0.11, 0.00], posterior inclusion probability = 0.40, inclusion Bayes factor = 0.65).

**Table 11:** Posterior coefficient summaries for the main Bayesian model predicting perceived stress (PSS-10) from group membership and coping styles.

Coefficient	Mean	SD	P(incl)	P(incl|data)	BF_inclusion_	95% credible interval	
						Lower	Upper
Intercept	14.57	0.29	1	1	1	14.02	15.1
Group: Civilian vs. Special Forces	0.57	0.95	0.5	0.38	0.61	−0.01	3
Group: Regular Army vs. Special Forces	0.23	0.69	0.5	0.23	0.31	−0.56	2.36
Group: WOT vs. Special Forces	0.98	1.08	0.5	0.58	1.39	0	3.13
SSZ - task-oriented style	−0.03	0.04	0.5	0.4	0.65	−0.11	0
SSE - emotion-oriented style	0.3	0.03	0.5	1	7.3E+12	0.24	0.37
SSU - avoidance-oriented style	0	0.02	0.5	0.18	0.21	−0.05	0.01

M, posterior mean; SD, posterior standard deviation; P(incl), prior inclusion probability; P(incl|data), posterior inclusion probability; BF_incl_, inclusion Bayes factor; CI, credible interval.

Evidence for group differences was inconclusive. The posterior means for all three comparisons with Special Forces were positive, corresponding to a lower mean PSS-10 score in this group; however, none of the comparisons received conclusive support: Territorial Defence Forces versus Special Forces (posterior mean = 0.98, SD = 1.08, 95% credible interval [0.00, 3.13], posterior inclusion probability = 0.58, inclusion Bayes factor = 1.39), civilians versus Special Forces (posterior mean = 0.57, SD = 0.95, 95% credible interval [−0.01, 3.00], posterior inclusion probability = 0.38, inclusion Bayes factor = 0.61), and Regular Army personnel versus Special Forces (posterior mean = 0.23, SD = 0.69, 95% credible interval [−0.56, 2.36], posterior inclusion probability = 0.23, inclusion Bayes factor = 0.31). Thus, after accounting for coping styles, the data provided no conclusive evidence either for or against independent differences between the groups.

The original exploratory coping model included group membership, SSZ, SSE, SSU, ACZ, and PKT (Table 12). Emotion-oriented coping had a positive coefficient (B = 0.31, β = 0.58; *p* < 0.001). Task-oriented coping had a negative coefficient but did not meet the significance threshold (β = −0.12; *p* = 0.062). The coefficients for avoidance-oriented coping and its components were not statistically significant in this specification. Because the avoidance total and its components overlap, these coefficients are conditional on closely related scores and cannot be interpreted as independent contributions of distinct coping constructs. In particular, non-significant coefficients do not demonstrate an absence of an association with perceived stress.

**Table 12:** Original exploratory linear regression model of perceived stress (PSS-10) including group membership, coping styles, and avoidance components.

Variable	B	SE	β	*p*	η^2^p
Group: Civilian vs. Special Forces	2.08	0.93	0.42	0.026	0.05
Group: Regular Army vs. Special Forces	2.52	0.87	0.51	0.004	
Group: WOT vs. Special Forces	1.71	0.92	0.34	0.065	
SSZ - task-oriented style	−0.07	0.04	−0.12	0.062	0.02
SSE - emotion-oriented style	0.31	0.04	0.58	< 0.001	0.29
SSU - avoidance-oriented style	−0.07	0.19	−0.12	0.721	0.00
ACZ - engagement in substitute activities	−0.06	0.22	−0.07	0.786	0.00
PKT - seeking social contacts	0.21	0.23	0.16	0.357	0.00

Linear regression model (OLS). R^2^ = 0.40. This is the original overlapping specification, not a refitted model with non-overlapping predictors.

η^2^p - partial eta squared effect size (0.01 = small, 0.06 = medium, 0.14 = large effect).

*p* < 0.05 indicates statistical significance. SSU, ACZ, and PKT overlap structurally; their coefficients are conditional on the other included scores and should not be interpreted as separable coping effects.

The sensitivity model, in which the overall avoidance-oriented coping score was replaced with its two components (Table 13), yielded consistent results. Emotion-oriented coping remained the only predictor with decisive evidence for inclusion (M = 0.31, SD = 0.04, 95% CI [0.23, 0.38], P(incl|data) = 1.00, BF_inclusion > 10^12^), and its estimated coefficient and credible interval were nearly identical to those obtained in the main model. The data did not favor the inclusion of either component of avoidance-oriented coping (ACZ: M = −0.04, SD = 0.06, 95% CI [−0.19, 0.00], BF_inclusion = 0.57; PKT: M = 0.01, SD = 0.05, 95% CI [−0.03, 0.17], BF_inclusion = 0.24). The intercept (14.57) and group coefficients (WOT = 0.97, civilian group = 0.57, Regular Army = 0.24) remained virtually unchanged relative to the main model. After SSU was omitted, only the posterior inclusion probability for SSZ increased slightly, from 0.40 to 0.47, while its inclusion Bayes factor increased from 0.65 to 0.87; these values still did not provide evidence in favor of its inclusion.

**Table 13:** Posterior coefficient summaries for the Bayesian sensitivity model predicting perceived stress (PSS-10) from group membership, task- and emotion-oriented coping, and the components of avoidance-oriented coping.

Coefficient	Mean	SD	P(incl)	P(incl|data)	BF_inclusion_	95% credible interval	
						Lower	Upper
Intercept	14.57	0.29	1	1	1	13.99	15.13
Group: Civilian vs. Special Forces	0.57	0.95	0.5	0.38	0.62	−0.05	2.83
Group: Regular Army vs. Special Forces	0.24	0.71	0.5	0.25	0.32	−0.37	2.14
Group: WOT vs. Special Forces	0.97	1.08	0.5	0.58	1.4	−0.03	3.06
SSZ - task-oriented style	−0.03	0.04	0.5	0.47	0.87	−0.13	0
SSE - emotion-oriented style	0.31	0.04	0.5	1	8.7E+12	0.23	0.38
ACZ - engagement in substitute activities	−0.04	0.06	0.5	0.36	0.57	−0.19	0
PKT - seeking social contacts	0.01	0.05	0.5	0.19	0.24	−0.03	0.17

M, posterior mean; SD, posterior standard deviation; P(incl), prior inclusion probability; P(incl|data), posterior inclusion probability; BF_incl_, inclusion Bayes factor; CI, credible interval.

The consistency between the two parameterizations indicates that the conclusions concerning emotion-oriented coping do not depend on how avoidance-oriented coping was entered into the model. At the same time, neither parameterization provides evidence that avoidance-oriented coping—whether represented by its overall score or by its components—makes an independent contribution to explaining perceived stress after accounting for the remaining predictors.

## Discussion

### Main findings

This study compared personality traits, coping styles, and perceived stress across three military formations and a civilian comparison group. The clearest descriptive pattern in perceived stress was a lower mean in Special Forces (M = 11.16; SD = 4.75) than in the other three groups. Special Forces also had higher conscientiousness than all other groups, lower neuroticism than civilians, and more frequent task-oriented coping than civilians. Emotion-oriented coping was lower in Special Forces than in civilians and WOT. Neuroticism and emotion-oriented coping showed the strongest positive bivariate associations with perceived stress within their respective domains. These findings characterize the sampled groups; they do not identify effects of military service, selection, or training.

Considering all four groups, the results do not suggest a simple distinction between military personnel and civilians. Civilians had higher neuroticism than Special Forces and the Regular Army, and lower conscientiousness than all three military groups. Regular Army and WOT participants had relatively similar mean perceived stress scores to civilians. Differences in coping were specific to selected pairwise comparisons rather than universal differences between soldiers and civilians. The inclusion of the civilian group therefore helps distinguish formation-specific patterns from patterns shared across the military groups, without implying that the groups are otherwise comparable or statistically equivalent.

### Personality traits and perceived stress

In the present study, the Five-Factor Model was used to describe the personality characteristics of participants. This model encompasses five basic dimensions of personality: extraversion, agreeableness, conscientiousness, neuroticism, and openness to experience. Previous research has highlighted the relevance of conscientiousness, neuroticism, and extraversion to psychological functioning under stressful conditions. Neuroticism is associated with a greater tendency to experience negative emotions and emotional reactivity, as well as with higher levels of perceived stress, whereas extraversion has more often been associated with more favorable psychological functioning (18, 19).

Differences in conscientiousness and neuroticism should be interpreted as differences in questionnaire scores rather than evidence of superior functioning in any group. Higher conscientiousness and lower neuroticism have been discussed in relation to self-control and emotional reactivity (20), and personality has been examined in relation to military decision-making under simulated operational stress (21). These findings provide context for the present associations, but decision-making, operational performance, and the effectiveness of self-regulation were not measured in this sample.

Published military studies also provide context for the observed association between neuroticism and perceived stress. Melkevik et al. (22) reported an association between neuroticism and post-traumatic stress symptoms in deployed soldiers. Huijzer et al. (23) described lower neuroticism and higher conscientiousness among experienced Dutch special forces operators compared with civilians and unsuccessful candidates. These findings should not be treated as interchangeable with the present PSS-10 results, and they do not establish whether selection, subsequent training, or operational experience explains the group differences in this sample.

Among the personality traits assessed here, neuroticism showed the strongest positive bivariate association with perceived stress (*r* = 0.59; *p* < 0.001). Kocjan et al. (19) also reported a prominent association between neuroticism and psychological functioning during the COVID-19 pandemic, while other work has linked neuroticism with anxiety and depression (24, 25). The present findings concern perceived stress rather than psychiatric diagnoses, and do not establish a temporal or causal pathway between these constructs.

Prospective research has examined personality traits in relation to subsequent psychological distress among soldiers (26), and longitudinal studies have considered changes in personality during combat-related stress or special operations selection (27, 28). Such findings support investigating professional trajectories over time, but cannot supply the missing individual histories in the present sample. Pre-existing selection-related characteristics and differences associated with age, training, or accumulated service experience remain alternative explanations that this study cannot separate.

The higher conscientiousness observed in Special Forces is also relevant to literature on personality and task performance. Research has examined personality composition in teams (29), associations of conscientiousness with academic and military performance during training (30), and conscientiousness in occupational functioning more generally (31). However, no performance outcome was measured in the present study. The between-group difference in conscientiousness therefore does not validate a selection criterion or demonstrate an advantage in operational performance.

### Perceived stress across the four comparison groups

Mean perceived stress was lower in Special Forces (11.16 ± 4.75) than in civilians (16.04 ± 4.84), the Regular Army (15.31 ± 4.92), and WOT (14.98 ± 4.48). This pattern is not a progressive gradient across the military formations, nor a uniform military–civilian contrast. The relative similarity of the latter three means does not establish equivalence. Furthermore, the use of raw scores without normative classification permits comparisons within this study, but not a conclusion that Special Forces participants had exceptionally low stress relative to the Polish population.

Formation membership is an observed grouping characteristic rather than an assigned exposure. The military groups may differ in age, selection-related characteristics, professional trajectories, training, duties, and exposure to stressful events. Because these dimensions were not comprehensively characterized, the lower mean PSS-10 score in Special Forces cannot be attributed to selection, training, combat experience, or an acquired response to repeated stress. These possibilities remain hypotheses for longitudinal studies with measurements before and during service.

The civilian comparison likewise requires a restricted interpretation. Civilian occupations, work demands, and potential sources of stress were not sufficiently characterized to establish occupational or exposure matching. Lack of military service does not imply lack of stressful experiences. Thus, the comparison concerns the four sampled groups and does not identify the psychological effect of entering military service or contrast inherently high-stress and low-stress occupations.

Studies in other occupations illustrate the relevance of professional context without providing a substitute control group. Research among marine engineers has examined associations between occupational rank, personality traits, and perceived stress (32). Other maritime studies have compared work-related stress in students and officers (33) and examined personality in relation to quality of life (34). These findings provide context for future comparisons with well-characterized occupational groups; differences in populations, instruments, and working conditions prevent direct transfer of their interpretations to the present sample.

### Coping styles and perceived stress

The coping results were specific to the comparisons supported by the post-hoc analyses: Special Forces reported more frequent task-oriented coping than civilians and lower emotion-oriented coping than civilians and WOT. Across the pooled sample, task-oriented coping was negatively associated with perceived stress (*r* = −0.25), whereas emotion-oriented coping showed a positive association (*r* = 0.60). These findings describe co-occurrence, not evidence that a coping style directly reduces or increases stress. The pooled correlations also do not establish that the same relationships hold separately within every group.

Research on acute military stress has examined associations of coping style with coping self-efficacy and appraisal emotions (35). Such work is relevant to understanding why coping measures may be associated with perceived stress, but the present study did not assess responses during an acute combat task or operational effectiveness.

The obtained data are also consistent with the systematic review conducted by Kirkham and colleagues, which indicated that task-oriented coping strategies and effective emotion regulation are associated with more favorable psychological outcomes among military personnel, including lower levels of PTSD, depression, and psychological exhaustion. At the same time, the authors reported associations between emotion-oriented and avoidance-oriented coping styles and greater psychological difficulties under conditions of chronic stress (11). These findings provide a broader context for the associations observed in the present study, but they do not establish causal relationships between coping styles and psychological outcomes.

Psychological resources may also be related to the choice of coping strategies. Studies by Netczuk-Gwoździewicz and Gołębiowski (36) demonstrated that soldiers with a higher sense of coherence more frequently used a task-oriented coping style, whereas individuals with a greater focus on emotions more often reported avoidance-oriented behaviors, including substitute activities. These findings provide additional context for the relationships between coping styles and perceived stress observed in the present study.

### Interpretation of the coping regression

The regression results require an additional distinction between the avoidance-oriented total (SSU) and its components, engagement in substitute activities (ACZ) and seeking social contacts (PKT). Their correlations with the avoidance total were 0.89 and 0.71, respectively. Entering the total and its components simultaneously means that a component coefficient is conditional on the total and the other scores. It is therefore not a straightforward estimate of the component’s association with perceived stress. The structural overlap can inflate coefficient uncertainty and make estimates sensitive to the predictor set.

For this reason, the original coping regressions are treated as exploratory and are not used to infer distinct effects or an absence of associations for avoidance coping and its components. Bayesian model averaging does not make the overlapping scores conceptually independent. A specification using the three main CISS styles and a separate specification replacing SSU with ACZ and PKT would address the total and its components without entering them together. The present tables do not report estimates from those alternative specifications, and the strength or stability of the corresponding coefficients cannot be assumed.

### Practical implications

The observed associations identify questions for further research on stress-management support, particularly the relationships of neuroticism and coping styles with perceived stress. They do not establish the effectiveness of an intervention, validate psychological selection criteria, or justify judging suitability for military service from these measures alone. Future longitudinal and intervention studies could evaluate whether changes in coping are accompanied by changes in perceived stress and whether associations differ across adequately characterized service and civilian contexts.

### Study limitations

The cross-sectional design limits the study to differences and associations measured at one time point. It does not identify the direction of associations between personality traits, coping styles, and perceived stress, or distinguish pre-existing differences from changes associated with service. The findings therefore cannot establish effects of selection, training, operational experience, or specific coping styles.

The sample was purposively recruited, included men only, and contained unequal group sizes, with 37 Special Forces participants. These features limit generalizability beyond the sampled participants and to female personnel. Self-report measures are also susceptible to reporting bias and social desirability; shared measurement methods may contribute to observed associations. The pooled analyses do not establish that the same associations occur within each group.

Incomplete demographic and professional characterization is a central limitation. The age eligibility interval of 18–50 years does not describe the age distribution of each group or establish age matching, and age was not included as a covariate. Consequently, the findings cannot be considered independent of age. Individual data on length of service, combat experience, and previous traumatic experiences were not collected, and selection history, training exposure, and professional trajectories were not comprehensively characterized. Their contributions cannot be separated from formation membership. Civilian occupations and sources of stress were also insufficiently characterized; the civilian group is therefore a non-military reference, not an established demographically, occupationally, or exposure-matched control group. Accordingly, observed differences should be interpreted as differences between the sampled groups, not as effects of formation membership or military service.

The original coping regression simultaneously included the avoidance-oriented total and its components. This structural overlap limits interpretation of the individual coefficients and Bayesian inclusion statistics. The tables do not provide results from non-overlapping alternative specifications, and the extent of coefficient stability across such models has not been established.

The psychological instruments describe personality traits, coping styles, and perceived stress rather than objectively measured stressor exposure. Biological stress responses and operational performance were not measured. The use of raw scores without age-specific normative classification also precludes conclusions about whether scores were low, average, or high relative to the Polish general population. Psychological resilience was not measured: PSS-10 scores indicate perceived stress during the preceding 4 weeks and cannot establish maintenance or recovery of functioning after adversity.

## Conclusion

In this cross-sectional sample of men, perceived stress was associated with personality traits and coping styles. Special Forces had a lower mean PSS-10 score than the other three groups, whose means were relatively similar, and higher conscientiousness than all other groups. Neuroticism and emotion-oriented coping showed the strongest positive bivariate associations with perceived stress within their respective domains. The pattern was not a uniform distinction between military and civilian participants. These findings describe the sampled groups and do not identify effects of selection, training, operational experience, or particular coping styles. Longitudinal studies with better-characterized comparison groups are needed to examine these relationships and evaluate their implications for stress-management support.

## Data Availability

The raw data supporting the conclusions of this article will be made available by the authors, without undue reservation.
